# Association between annular eccentricity and initial post-deployment paravalvular leak before corrective maneuvers in bicuspid aortic valve patients undergoing self-expanding transcatheter aortic valve replacement: a single-center retrospective study

**DOI:** 10.3389/fcvm.2026.1909489

**Published:** 2026-08-05

**Authors:** Chen Cui, Zhanjun Qu, Ze Zhao, Weili Liu, Lei Jiang

**Affiliations:** 1 Department of Cardiovascular Surgery, Affiliated Hospital of Qingdao University, Qingdao, China; 2 Department of Radiology, Qingdao Eighth People’s Hospital, Qingdao, China; 3 Interventional Operation Room, Affiliated Hospital of Qingdao University, Qingdao, China

**Keywords:** bicuspid aortic valve, calcification, eccentricity index, paravalvular leak, transcatheter aortic valve replacement, VenusA valve

## Abstract

**Objective:**

The relationship between annular eccentricity and paravalvular leak (PVL) after transcatheter aortic valve replacement (TAVR) in bicuspid aortic valve (BAV) patients remains insufficiently defined, particularly for Chinese self-expanding valve platforms. This study evaluated the association between the CT-derived eccentricity index (EI) and initial post-deployment moderate-or-greater PVL after self-expanding TAVR in BAV patients.

**Methods:**

We retrospectively analyzed 194 consecutive BAV patients who underwent TAVR with the first-generation self-expanding VenusA valve. The primary endpoint was moderate-or-greater PVL assessed immediately after valve release and before corrective maneuvers such as post-dilatation. EI was measured by two independent observers with excellent interobserver agreement (ICC = 0.943). Multivariable logistic regression, ROC analysis, sensitivity analyses, and bootstrap internal validation were performed. NRI and IDI were analyzed as secondary measures of incremental information.

**Results:**

Initial post-deployment moderate-or-greater PVL occurred in 62 patients (32.0%). Although EI was modestly higher in patients with moderate-or-greater PVL than in those with no/mild PVL (median 0.23 [IQR 0.19–0.26] vs. 0.20 [0.17–0.25]; *P* = 0.005), its stand-alone discrimination was poor (AUC = 0.625; 95% CI 0.539–0.711). After adjustment for clinical and anatomical covariates, EI ≥0.19 remained independently associated with moderate-or-greater PVL (OR = 2.99; 95% CI 1.44–6.23; *P* = 0.0034), whereas semi-quantitative leaflet calcification and raphe calcification were not. Adding EI increased the C-index only marginally (apparent 0.572 → 0.666; optimism-corrected 0.609), indicating limited overall model discrimination. Predischarge transesophageal echocardiography was available for all 194 patients: moderate-or-greater PVL fell from 62 patients (32.0%) at deployment to only 6 (3.1%) at predischarge, and 57 of the 62 patients with initial moderate-or-greater PVL (91.9%) had regressed to mild or less (paired Wilcoxon signed-rank, *P* < 0.001), underscoring that initial post-deployment PVL does not equate to clinically relevant residual PVL.

**Conclusion:**

In BAV patients undergoing TAVR with the first-generation self-expanding VenusA valve, EI ≥0.19 was independently associated with initial post-deployment moderate-or-greater PVL, but its stand-alone and model-level discriminative performance were limited. EI should be considered a supplementary dimension in integrated preprocedural risk assessment rather than an isolated decision-making criterion.

## 1 Introduction

The bicuspid aortic valve (BAV) is the most common congenital cardiac valve malformation, with a prevalence of approximately 1%–2% in the general population (1). Compared with patients who have a tricuspid aortic valve (TAV), patients with BAV typically present at a younger age and exhibit heavier valvular calcification and a more elliptical, rather than circular, annular geometry; these anatomical differences pose unique challenges for transcatheter aortic valve replacement (TAVR) (2, 3). Research on BAV-TAVR has advanced considerably in recent years, yet the predictive value of annular asymmetry for postprocedural paravalvular leak remains controversial, particularly for Chinese self-expanding valve platforms, for which data are largely lacking.

Paravalvular leak (PVL) is one of the most common complications after TAVR, and moderate-or-greater PVL is independently associated with increased long-term mortality (4, 5). In the era of self-expanding valves, although newer-generation valve designs (e.g., Evolut PRO/PRO+ and VenusA-PRO) have substantially reduced the incidence of PVL by adding an outer sealing skirt, the risk of moderate-or-greater PVL in patients with BAV remains significantly higher than in patients with TAV, owing to anatomical features such as annular asymmetry and heterogeneous calcification distribution (6, 7). A recent international multicenter registry of 946 patients with Sievers type 1 BAV reported an incidence of moderate-or-greater PVL of approximately 4% after TAVR with newer-generation transcatheter valves, and identified the self-expanding valve as a significant predictor of PVL (OR = 9.01) (8).

The eccentricity index (EI) is a commonly used CT-derived parameter that quantifies the degree of elliptical annular geometry. It is defined as EI = 1−(minimum annular diameter/maximum annular diameter), ranging from 0 to 1, with higher values indicating a more irregular annulus. With advances in CT imaging, precise assessment of three-dimensional annular morphology in patients with BAV has received increasing attention (9). Previous studies have provided preliminary reports of an association between EI and post-TAVR PVL in Western populations (10, 11); however, these studies predominantly involved patients with a tricuspid aortic valve and largely used balloon-expandable valves. In patients with BAV—and particularly for the Chinese domestically developed self-expanding VenusA valve platform—the predictive value of EI for initial post-deployment PVL (before corrective maneuvers) has not been systematically validated. In addition, leaflet calcification severity and raphe calcification have been regarded as contributing factors for PVL (12, 13), but their independent predictive role in BAV populations is likewise debated. A meta-analysis of 24 studies comprising 6,846 patients indicated that the quantity and location of calcification have predictive value for PVL, although this association is attenuated by newer valves equipped with a sealing skirt (14).

Over the past two decades, TAVR has evolved rapidly and has been increasingly adopted in China (18). The VenusA valve (Venus Medtech, Hangzhou, China) is a first-generation, domestically developed self-expanding TAVR valve with porcine pericardial leaflets and a nitinol stent platform. Previous studies have supported the feasibility and safety of this valve in Chinese patients, including those with BAV (15, 16). Newer VenusA-Pro/Plus systems have incorporated retrievability and sealing-skirt features and have been associated with lower rates of moderate-or-greater PVL (17). However, evidence remains limited regarding anatomical predictors of PVL with the first-generation skirtless VenusA valve in BAV patients. This study therefore evaluated the association between annular EI and initial post-deployment moderate-or-greater PVL in a Chinese BAV cohort treated with first-generation self-expanding VenusA valves. We deliberately focused on the pre-correction time point because, from the operator's standpoint, final and long-term PVL are largely non-modifiable once the patient leaves the catheterization laboratory, whereas the moments immediately after valve release constitute the only window in which PVL can be actively addressed through post-dilatation, repositioning, or valve-in-valve. Our aim was thus twofold: to test whether EI is independently associated with this intraprocedurally modifiable endpoint, and—equally—to delineate the boundaries of its usefulness, positioning EI as a supplementary anatomical dimension rather than a stand-alone predictive or decision-making tool.

## 2 Materials and methods

### 2.1 Study design and participants

This was a single-center, retrospective, observational study that consecutively enrolled 194 patients with a bicuspid aortic valve (BAV) who underwent TAVR with the self-expanding VenusA valve at the Department of Cardiovascular Surgery, Affiliated Hospital of Qingdao University, between January 2018 and December 2025. The inclusion criteria were: (1) severe aortic stenosis confirmed by echocardiography; (2) a bicuspid aortic valve confirmed by preprocedural CT (Sievers type 0, 1, or 2); and (3) treatment by TAVR with the VenusA valve. The exclusion criteria were: (1) prior valve replacement; (2) severe renal insufficiency (eGFR < 30 mL/min/1.73 m^2^); and (3) incomplete clinical data.

This study was approved by the Ethics Committee of Affiliated Hospital of Qingdao University (ethics approval number: QYFY-WZLL-50271), with a waiver of informed consent. All study procedures were conducted in accordance with the Declaration of Helsinki and its subsequent amendments.

### 2.2 CT acquisition and parameter measurement

Given that the 2025 ESC/EACTS Guidelines for the management of valvular heart disease emphasize the need for comprehensive imaging assessment before TAVR in patients with BAV (19), all patients in this study underwent ECG-gated CT angiography before the procedure. The maximum and minimum annular diameters, area, and perimeter were measured from the archived ECG-gated CT images by two independent observers using 3mensio Structural Heart post-processing software (Pie Medical Imaging); both observers were blinded to the patients' postprocedural PVL outcome. The eccentricity index (EI) was calculated as EI = 1−(minimum diameter/maximum diameter). Interobserver agreement for EI was assessed using a two-way random-effects, absolute-agreement, single-measurement intraclass correlation coefficient (ICC); the ICC was 0.943 (95% CI 0.917–0.960), indicating excellent interobserver agreement (20). Leaflet calcification severity was assessed using the visual semi-quantitative grading recommended by the 2019 SCCT expert consensus document (21): grade 0, no calcification; grade 1, mild calcification (scattered spots without a tendency to coalesce); grade 2, moderate calcification (calcific spots coalescing into patches involving part of a leaflet); and grade 3, severe calcification (large nodular calcification extending to the leaflet margin). Moderate-or-greater calcification was defined as a calcification grade ≥2. The calcification grading workflow was as follows. The initial assessment was performed jointly by the periprocedural cardiac intervention team (including the operating surgeon, the imaging physician, and the clinical support staff of the valve manufacturer) during preprocedural CT planning for TAVR, based on multiplanar reformatting (MPR) and maximum-intensity projection (MIP) images in 3mensio Structural Heart software, and was used directly for clinical procedural strategy. At the time of data collection for this study, an imaging physician who had not participated in the initial assessment and who was blinded to the patients’ postprocedural PVL outcome independently retrieved the archived 3mensio reports and MIP images and performed a second, confirmatory review of the calcification severity and raphe calcification for all patients. Interobserver agreement for calcification grading was assessed using a quadratic weighted *κ*, which was 0.967 (95% CI 0.949–0.982), indicating almost perfect interobserver agreement. It should be noted that, owing to constraints on the availability of retrospective data, calcification was assessed by visual semi-quantitative grading rather than by quantitative CT calcium volume; this is one of the limitations of the present study and is discussed further in the Discussion. The Sievers classification of the bicuspid aortic valve was determined by preprocedural CT assessment and categorized as type 0 (no raphe), type 1 (one raphe), or type 2 (two raphes); given the very small number of type 2 cases, types 1 and 2 were combined as “raphe-containing type (non-type 0)” for statistical analysis.

### 2.3 TAVR procedure

All procedures were performed in the interventional operating room by the interventional team of the Department of Cardiovascular Surgery, Affiliated Hospital of Qingdao University, under general anesthesia via transfemoral access. Valve-size selection was based on the CT-measured annular area and perimeter, with oversizing calculated according to the manufacturer's recommendations. The oversizing ratio was defined as (nominal valve area/CT-measured annular area−1) × 100%. All procedures were completed under intraprocedural aortic root digital subtraction angiography (DSA) guidance. Transesophageal echocardiography (TEE) was routinely performed during the procedure to assess valve position, function, and paravalvular leak. Because of the constraints of retrospective data, procedural variables such as implantation depth were not included in this study, which may be an important reason for the limited predictive performance of the model. The decision to perform balloon predilatation and post-dilatation was made intraprocedurally on the basis of the CT measurements. All 194 patients in this cohort received the first-generation self-expanding VenusA valve (Venus Medtech, Hangzhou), with nominal sizes of L23, L26, L29, and L32 (the number denotes the nominal inflow diameter of the valve, in mm). All patients met the criteria for device implantation success, and none required implantation of a second valve.

### 2.4 PVL assessment

The primary endpoint was initial post-deployment PVL, defined as moderate-or-greater PVL graded immediately after valve release and before any corrective maneuver (post-dilatation or valve-in-valve implantation). PVL was graded at the time of the procedure by the TAVR team's imaging physician together with the operating physician using both intraprocedural aortic root DSA and TEE, according to VARC-3 and ASE recommendations (0 = none/trace, 1 = mild, 2 = moderate, 3 = severe), with moderate-or-greater PVL defined as grade ≥2. The physicians grading intraprocedural PVL were not involved in, and were blinded to, the CT-derived EI, which was measured separately and offline by two independent observers (see Section 2.2). When DSA and TEE were discordant, the TEE grade was adopted as the reference, in accordance with VARC-3 recommendations. In addition, predischarge PVL was assessed by transesophageal echocardiography before hospital discharge in all patients, and post-discharge PVL was assessed by follow-up echocardiography. All echocardiographic studies were reported by senior echocardiographers of the TAVR team. This time point was chosen because it best reflects the annulus–stent geometric mismatch before therapeutic correction and delayed nitinol-frame expansion. The interpretation of our results is therefore confined to initial post-deployment PVL and should not be extrapolated to final procedural, predischarge, or long-term PVL.

### 2.5 Statistical analysis

Statistical analyses were performed using Python 3.13. Continuous variables are presented as mean ± SD or median (IQR) according to distribution, and categorical variables as *n* (%). Between-group comparisons used the independent-samples *t*-test, Mann–Whitney *U*-test, chi-square test, or Fisher exact test, as appropriate. Interobserver agreement was assessed by the ICC for EI and by weighted kappa for calcification grading. Balloon post-dilatation was not entered into the predictive model, because it is a remedial maneuver performed in response to—and temporally after—the study endpoint (initial post-deployment PVL, assessed after valve release but before post-dilatation), and thus represents a clinical reaction to, rather than a predictor of, the outcome.

The stand-alone discrimination of EI was evaluated by ROC analysis. EI was analyzed primarily as a continuous variable (per 0.1 increase), which is more robust and does not depend on a threshold. The EI ≥0.19 cutoff was derived from the Youden index within the same cohort and is therefore regarded as exploratory and hypothesis-generating only, requiring external validation before any clinical use; it is not presented as an established clinical threshold. The primary multivariable logistic model included EI ≥0.19, moderate-or-greater calcification, raphe calcification, age ≥65 years, male sex, NYHA class ≥III, and BMI ≥25 kg/m^2^. Model robustness was examined in prespecified sensitivity analyses that additionally adjusted for oversizing ratio, annular size, annular area, Sievers classification, or balloon predilatation, and Firth penalized regression was used to address sparse-data bias. Internal validation and optimism correction used bootstrap resampling (1,000 replicates), and NRI and IDI were reported as secondary measures of incremental information. A two-sided *P* < 0.05 was considered statistically significant. Two additional prespecified sensitivity analyses were performed at the request of review: age modeled as a continuous variable (Supplementary Table S5) and exclusion of patients with missing BMI (Supplementary Table S6). Generative AI was used solely to assist with statistical coding and figure generation; it was not involved in data generation, measurement, or interpretation. All analyses are fully reproducible from the underlying dataset, which is available on reasonable request.

CT, echocardiographic, and initial post-deployment PVL data were complete for all 194 patients. Five bedridden patients with missing height/weight were conservatively coded as BMI <25 kg/m^2^ for the binary BMI analysis. Balloon predilatation status, available in 181 patients, was used in sensitivity analysis only.

## 3 Results

### 3.1 Baseline characteristics

**Figure 1:**
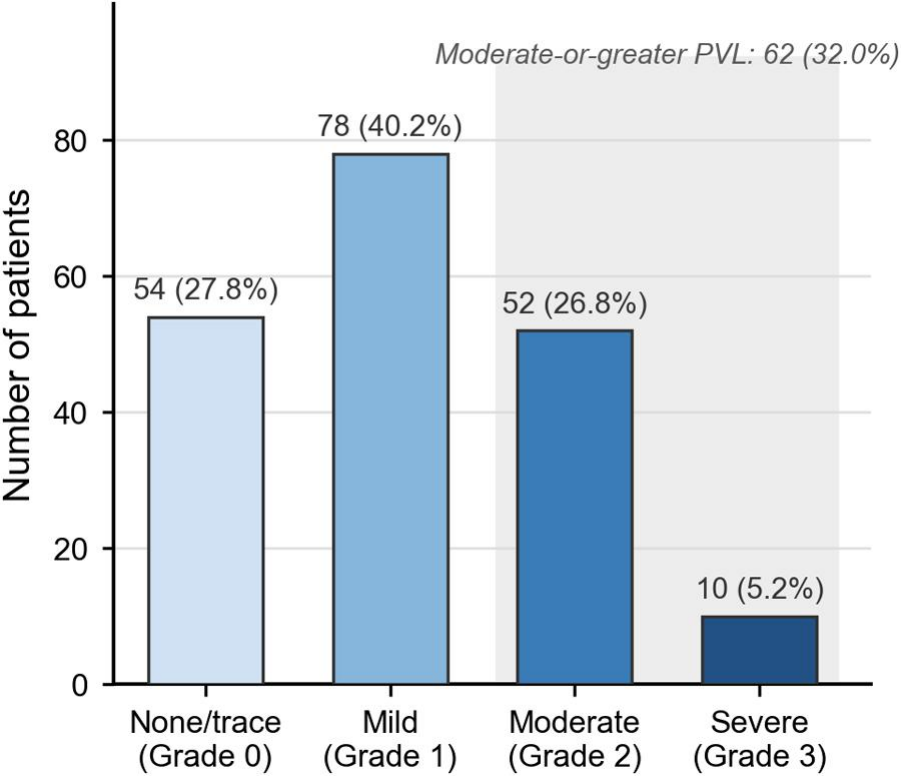
Distribution of initial post-deployment paravalvular leak (PVL) severity (*n* = 194). Bar chart showing the number and percentage of patients with none/trace (grade 0, *n* = 54, 27.8%), mild (grade 1, *n* = 78, 40.2%), moderate (grade 2, *n* = 52, 26.8%), and severe (grade 3, *n* = 10, 5.2%) PVL after transcatheter aortic valve replacement with the self-expanding VenusA valve in patients with a bicuspid aortic valve.

A total of 194 patients with BAV were enrolled; the mean age was 75.0 ± 7.6 years, and 99 (51.0%) were male. Initial post-deployment PVL (PVL grade ≥2) occurred in 62 patients (32.0%) (Figure 1). The two groups were well balanced with respect to demographic and clinical baseline characteristics—including age, sex, body mass index (BMI), and the burden of comorbidities such as hypertension, diabetes, and coronary artery disease (all *P* > 0.05; Table 1). Notably, the maximum annular diameter, minimum annular diameter, and annular area did not differ between groups (26.8 [24.6–29.0] vs. 26.6 [24.9–28.2] mm, *P* = 0.711; 20.7 [19.2–21.7] vs. 20.9 [19.6–22.5] mm, *P* = 0.193; and 437.5 [366.2–477.8] vs. 433.4 [377.4–493.5] mm^2^, *P* = 0.580, respectively). In contrast, the annular eccentricity index (EI)—which integrates these two diameters into a measure of shape rather than size—was significantly higher in the moderate-or-greater PVL group than in the no/mild PVL group (median 0.23 [IQR 0.19–0.26] vs. 0.20 [0.17–0.25]; *P* = 0.005), and EI ≥0.19 was more frequent in the moderate-or-greater PVL group (80.6% vs. 58.3%; *P* = 0.002). The proportion of patients aged ≥65 years was nominally higher in the moderate-or-greater PVL group (98.4% vs. 90.2%; *P* = 0.040); however, only 1 patient in this group was younger than 65 years (Supplementary Table S1). According to the Sievers classification, 143 patients (73.7%) were type 0, 49 (25.3%) were type 1, and 2 (1.0%) were type 2; the distribution of the raphe-containing type (type 1/2) did not differ significantly between the moderate-or-greater PVL group and the no/mild PVL group (25.8% in the moderate-or-greater PVL group vs. 26.5% in the no/mild PVL group; *P* = 1.000).

**Table 1:** Baseline, anatomical, and procedural characteristics of the overall cohort and stratified by initial post-deployment moderate-or-greater paravalvular leak (PVL).

Characteristic	Overall (*n* = 194)	No/mild PVL (*n* = 132)	Mod/severe PVL (*n* = 62)	*P*
Demographics				
Age, years, median (IQR)	75.0 (70.0–80.0)	75.0 (70.0–80.0)	75.0 (70.3–79.8)	0.445
Age ≥65, *n* (%)	180 (92.8)	119 (90.2)	61 (98.4)	0.040
Male, *n* (%)	99 (51.0)	68 (51.5)	31 (50.0)	0.878
BMI ≥25 kg/m^2^, *n* (%)	79 (40.7)	54 (40.9)	25 (40.3)	1.000
Hypertension, *n* (%)	113 (58.2)	81 (61.4)	32 (51.6)	0.215
Diabetes, *n* (%)	53 (27.3)	35 (26.5)	18 (29.0)	0.732
Coronary artery disease, *n* (%)	70 (36.1)	44 (33.3)	26 (41.9)	0.265
NYHA III–IV, *n* (%)	153 (78.9)	101 (76.5)	52 (83.9)	0.265
Anatomical/CT characteristics				
Annular area, mm^2^, median (IQR)	436.1 (374.0–491.5)	433.4 (377.4–493.5)	437.5 (366.2–477.8)	0.580
Maximum annular diameter, mm, median (IQR)	26.6 (24.9–28.5)	26.6 (24.9–28.2)	26.8 (24.6–29.0)	0.711
Minimum annular diameter, mm, median (IQR)	20.9 (19.5–22.3)	20.9 (19.6–22.5)	20.7 (19.2–21.7)	0.193
Eccentricity index, median (IQR)	0.21 (0.17–0.25)	0.20 (0.17–0.25)	0.23 (0.19–0.26)	0.005
EI ≥0.19, *n* (%)	127 (65.5)	77 (58.3)	50 (80.6)	0.002
Oversizing ratio, %, median (IQR)	11 (5–16)	11 (5–16)	11 (6–17)	0.540
Heavy (moderate–severe) calcification, *n* (%)	107 (55.2)	72 (54.5)	35 (56.5)	0.877
Raphe calcification, *n* (%)	44 (22.7)	30 (22.7)	14 (22.6)	1.000
Sievers classification, *n* (%)				1.000
Type 0	143 (73.7)	97 (73.5)	46 (74.2)	
Type 1	49 (25.3)	34 (25.8)	15 (24.2)	
Type 2	2 (1.0)	1 (0.8)	1 (1.6)	
Procedural characteristics				
Valve size, *n* (%)				0.848
L23	42 (21.6)	27 (20.5)	15 (24.2)	
L26	85 (43.8)	60 (45.5)	25 (40.3)	
L29	59 (30.4)	39 (29.5)	20 (32.3)	
L32	8 (4.1)	6 (4.5)	2 (3.2)	
Balloon pre-dilatation, *n* (%)^a^	154/181 (85.1)	102/121 (84.3)	52/60 (86.7)	0.825
Balloon post-dilatation, *n* (%)^a^^,^^b^	87/181 (48.1)	34/121 (28.1)	53/60 (88.3)	<0.001

Continuous variables are presented as median (interquartile range) and compared using the Mann–Whitney *U*-test; categorical variables are presented as *n* (%) and compared using Fisher's exact test. Sievers classification was dichotomized as type 0 versus type 1/2 for comparison (Fisher's exact test, *P* = 1.000).

a Pre- and post-dilatation data were available for 181 of 194 patients (no/mild PVL group, *n* = 121; moderate-or-greater PVL group, *n* = 60); the percentages and between-group comparisons for these two variables are based on these subgroups.

b Post-dilatation represents a corrective response to initial post-deployment PVL (reverse causality) and was therefore reported descriptively but not included in the predictive models. Five bedridden patients without measurable BMI were coded as BMI <25 kg/m^2^. BMI, body mass index; CT, computed tomography; EI, eccentricity index; IQR, interquartile range; NYHA, New York Heart Association; Mod/severe, moderate-or-greater; PVL, paravalvular leak.

### 3.2 ROC curve analysis

**Figure 2:**
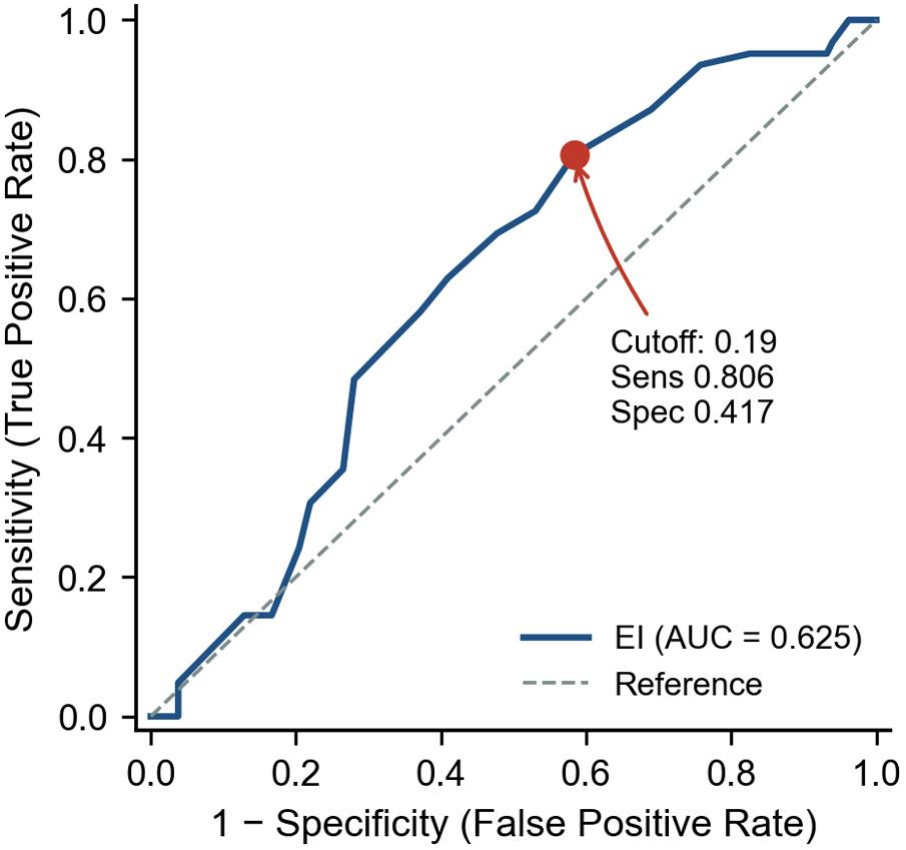
Receiver operating characteristic (ROC) curve of the eccentricity index (EI) for predicting moderate-or-greater paravalvular leak. The area under the curve (AUC) is 0.625. The optimal cutoff value was 0.19, yielding a sensitivity of 0.806 and a specificity of 0.417. The diagonal dashed line represents the reference line of no discrimination.

**Figure 3:**
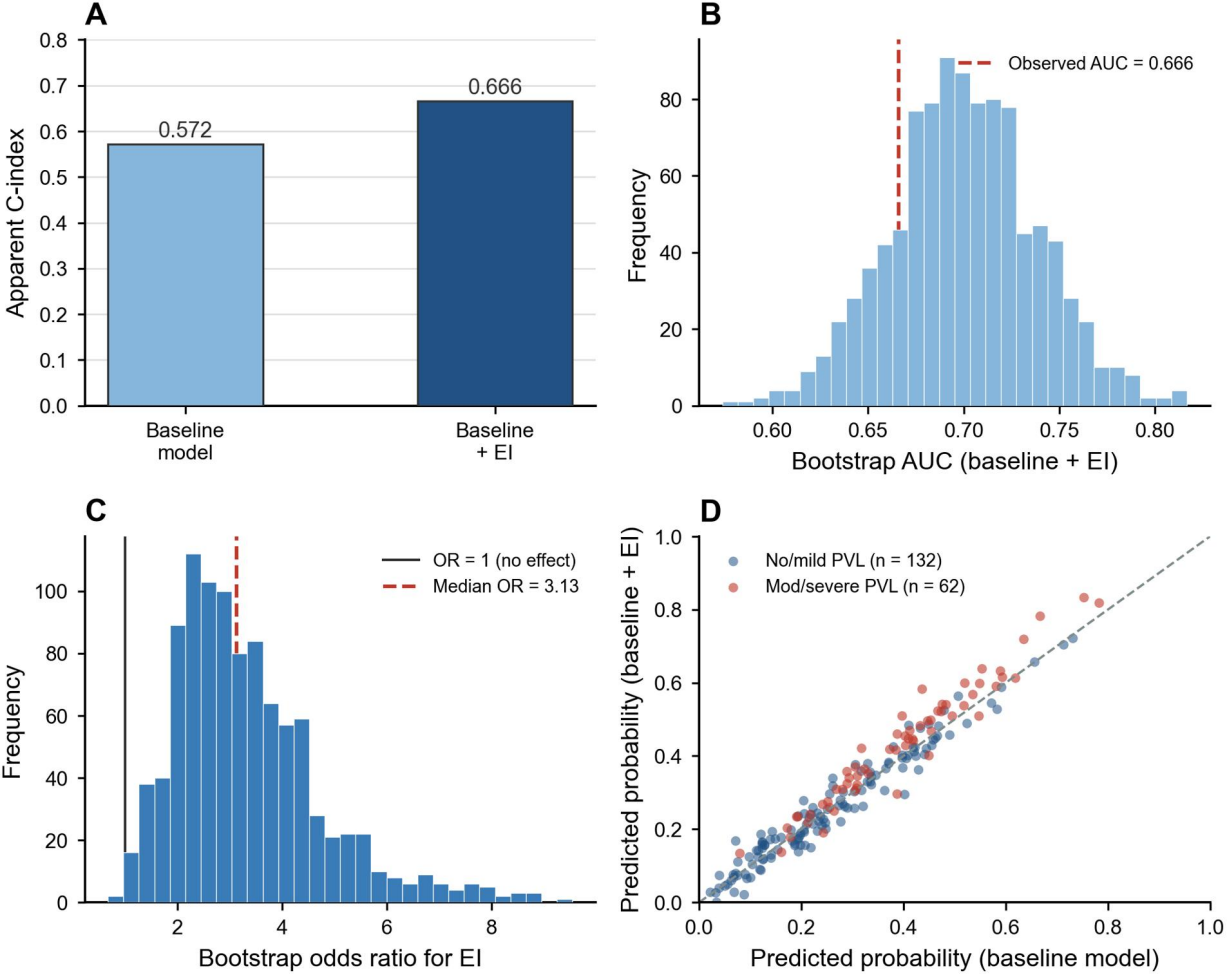
Incremental predictive value of the eccentricity index (EI) for moderate-or-greater paravalvular leak (PVL). **(A)** Apparent discrimination of the baseline model (C-index = 0.572) and the baseline + EI model (C-index = 0.666). **(B)** Bootstrap distribution of the area under the curve (AUC) for the new model (baseline + EI), with an observed AUC = 0.666 and a bootstrap mean = 0.700 (95% CI: 0.625–0.772). **(C)** Bootstrap distribution of the odds ratios (OR) for EI, with a median OR = 3.13 (95% CI: 1.46–7.21); this bootstrap median corresponds to the apparent multivariable point estimate of OR = 2.99 reported in Table 2. The reference line is shown at OR = 1 (no effect). **(D)** Scatter plot comparing the predicted probabilities of moderate-or-greater PVL between the baseline model and the new model, stratified by no/mild PVL (*n* = 132) and moderate-or-greater PVL (*n* = 62).

ROC curve analysis (Figure 2) showed that EI alone showed poor stand-alone discrimination for initial post-deployment moderate-or-greater PVL, with an AUC of 0.625 (95% CI 0.539–0.711). When EI was added to the multivariable baseline model, the apparent C-index increased from 0.572 to 0.666 (Figure 3), although bootstrap optimism correction reduced the C-index to 0.609. Thus, EI provided incremental information, but the overall discrimination of the model remained modest. As secondary measures of incremental information, adding EI to the baseline model yielded a continuous NRI of 0.446 (*P* = 0.002; driven mainly by improved classification among events, 61.3%) and an IDI of 0.040. We interpret these values with deliberate caution: NRI and IDI can suggest incremental value even when overall discrimination remains poor, and they are known to be optimistic and unstable in models with limited C-statistics such as ours. They are therefore reported only as exploratory, supportive evidence of a directionally consistent signal, and not as evidence of clinically meaningful predictive gain—consistent with the modest AUC (0.625) and optimism-corrected C-index (0.609).

**Table 2:** Multivariable logistic regression analysis for predictors of initial post-deployment moderate-or-greater paravalvular leak.

Variable	OR (95% CI)	Z value	*P* value
Eccentricity index ≥0.19	2.99 (1.44–6.23)	2.93	0.0034
Moderate-or-greater calcification	1.04 (0.53–2.03)	0.11	0.9101
Raphe calcification	0.93 (0.42–2.06)	−0.18	0.8577
Age ≥65 years	7.30 (0.91–58.39)	1.87	0.0611
Male sex	0.92 (0.47–1.78)	−0.26	0.7953
NYHA class ≥III	1.66 (0.72–3.80)	1.19	0.2334
BMI ≥25 kg/m^2^	0.99 (0.51–1.91)	−0.03	0.9765

Variables were selected *a priori* based on clinical relevance and anatomical plausibility, including EI ≥0.19, moderate-or-greater calcification, raphe calcification, age ≥65 years, male sex, NYHA class ≥III, and BMI ≥25 kg/m^2^. Odds ratios (OR) and 95% confidence intervals (CI) are reported (*n* = 194; events = 62). EI ≥0.19 was the only independent predictor of initial post-deployment moderate-or-greater PVL. BMI, body mass index; CI, confidence interval; EI, eccentricity index; NYHA, New York Heart Association; OR, odds ratio.

### 3.3 Multivariable logistic regression analysis

**Figure 4:**
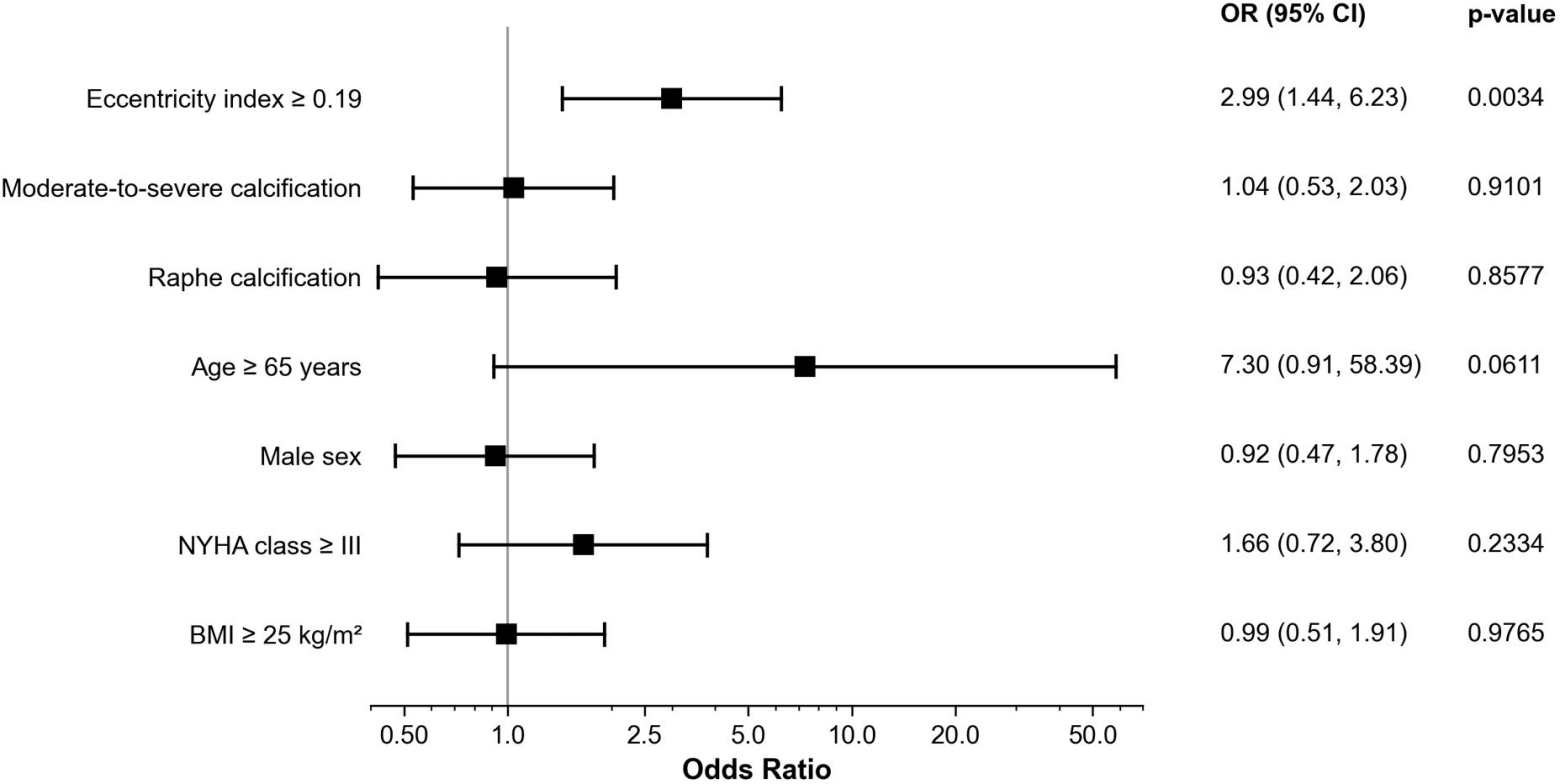
Forest plot of the multivariable logistic regression analysis for predictors of moderate-or-greater paravalvular leak (PVL). Odds ratios (OR) with 95% confidence intervals (CI) are shown for each predictor: body mass index ≥25 kg/m^2^ (OR = 0.99, 95% CI 0.51–1.91, *P* = 0.9765), NYHA class ≥III (OR = 1.66, 95% CI 0.72–3.80, *P* = 0.2334), male sex (OR = 0.92, 95% CI 0.47–1.78, *P* = 0.7953), age ≥65 years (OR = 7.30, 95% CI 0.91–58.39, *P* = 0.0611), raphe calcification (OR = 0.93, 95% CI 0.42–2.06, *P* = 0.8577), moderate-or-greater calcification (OR = 1.04, 95% CI 0.53–2.03, *P* = 0.9101), and eccentricity index ≥0.19 (OR = 2.99, 95% CI 1.44–6.23, *P* = 0.0034). EI ≥0.19 was the only statistically significant predictor. Analysis based on the self-expanding VenusA valve in patients with a bicuspid aortic valve (*n* = 194).

**Figure 5:**
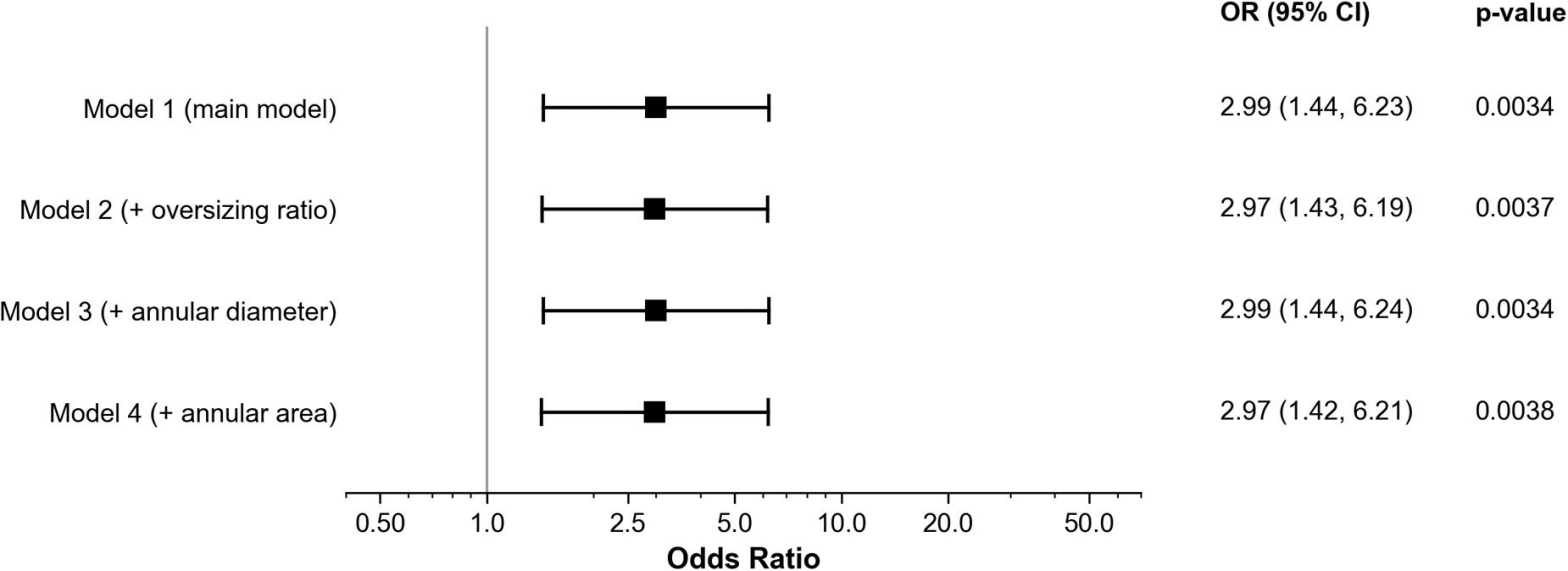
Sensitivity analysis showing the stability of the eccentricity index (EI ≥0.19) effect across multivariable models. Forest plot of the OR of EI for moderate-or-greater paravalvular leak across four models. Model 1 (main model, baseline covariates): OR = 2.99 (95% CI 1.44–6.23, *P* = 0.0034). Model 2 (Model 1 + oversizing ratio): OR = 2.97 (95% CI 1.43–6.19, *P* = 0.0037). Model 3 (Model 1 + annular diameter): OR = 2.99 (95% CI 1.44–6.24, *P* = 0.0034). Model 4 (Model 1 + annular area): OR = 2.97 (95% CI 1.42–6.21, *P* = 0.0038). The effect of EI remained stable and statistically significant across all models. Self-expanding VenusA valve, bicuspid aortic valve, *n* = 194.

The procedural characteristics of the groups are shown in Table 1. Neither valve size (L23/L26/L29/L32) nor the rate of balloon predilatation differed significantly between the groups (*P* = 0.848 and *P* = 0.825, respectively). Balloon post-dilatation was significantly more frequent in the initial post-deployment PVL group (88.3% vs. 28.1%; *P* < 0.001). The results of the multivariable logistic regression analysis are shown in Table 2 and Figure 4. After adjustment for confounders including age, sex, BMI, NYHA class, calcification severity, and raphe calcification, EI ≥0.19 was the only variable independently associated with initial post-deployment PVL in this model (OR = 2.99; 95% CI: 1.44–6.23; *P* = 0.0034); this should be interpreted as an independent association within the available covariate set rather than evidence that EI is the dominant or causal determinant of PVL.Moderate-or-greater calcification (OR = 1.04; 95% CI: 0.53–2.03; *P* = 0.910), raphe calcification (OR = 0.93; 95% CI: 0.42–2.06; *P* = 0.858), age ≥65 years (OR = 7.30; 95% CI: 0.91–58.39; *P* = 0.061), male sex (OR = 0.92; 95% CI: 0.47–1.78; *P* = 0.795), NYHA class ≥III (OR = 1.66; 95% CI: 0.72–3.80; *P* = 0.233), and BMI ≥25 kg/m^2^ (OR = 0.99; 95% CI: 0.51–1.91; *P* = 0.977) showed no significant association with moderate-or-greater PVL. The OR for age ≥65 years was numerically large but non-significant, with a very wide confidence interval (OR = 7.30; 95% CI 0.91–58.39; *P* = 0.061), reflecting sparse events in this subgroup (only 1 event occurred in patients aged < 65 years); this estimate was markedly attenuated after Firth penalized regression (Supplementary Table S1) and after modeling age continuously (Supplementary Table S5). In addition, when EI was entered as a continuous variable in the same multivariable model, each 0.1 increase in EI was associated with an approximately 87% increase in the risk of initial post-deployment PVL (OR = 1.87; 95% CI 1.08–3.25; *P* = 0.026) (Supplementary Table S3), consistent in direction with the binary (EI ≥0.19) result, indicating that this association did not depend on the choice of a particular cutoff. Bootstrap resampling (1,000 replicates) showed that the median of the Youden-optimal cutoff was 0.21 (95% CI 0.17–0.24), and the cutoff obtained from the original data (0.19) fell within this interval, indicating that the chosen cutoff was reasonably stable. When age was modeled as a continuous variable instead of the ≥65-year dichotomy, the association of EI ≥0.19 was essentially unchanged (OR = 2.97; 95% CI 1.43–6.16; *P* = 0.0034), while continuous age was not significant (OR = 1.02 per year; 95% CI 0.98–1.07; *P* = 0.29), confirming that the wide, unstable OR for dichotomized age reflected data sparsity rather than a true large effect (Supplementary Table S3). In a further sensitivity analysis excluding the five patients with missing BMI (*n* = 189; events = 59), EI ≥0.19 remained independently associated with the endpoint (OR = 2.81; 95% CI 1.34–5.89; *P* = 0.006), indicating that the conservative BMI coding did not affect the principal findings. In addition, in the sensitivity analysis that additionally adjusted for balloon predilatation on top of the primary model (*n* = 181; events = 60; 13 patients excluded because of missing predilatation status, of whom 2 had moderate-or-greater PVL), the association between EI ≥0.19 and initial post-deployment PVL was unchanged (OR = 3.00; 95% CI 1.42–6.33; *P* = 0.0039), and predilatation itself was not significantly associated with the outcome (OR = 1.31; 95% CI 0.47–3.64; *P* = 0.607) (Supplementary Table S4) (Figure 5).

Firth penalized regression was performed to address the sparse event distribution of the age ≥65 years variable (Supplementary Table S1). EI ≥0.19 remained independently associated with initial post-deployment moderate-or-greater PVL (OR = 2.82; 95% CI 1.38–5.79; *P* = 0.0046), whereas the OR for age ≥65 years shrank with a narrower confidence interval.

### 3.4 Follow-up outcomes

Predischarge transesophageal echocardiography was available for all 194 patients. Although initial post-deployment moderate-or-greater PVL occurred in 62 patients (32.0%), only 6 patients (3.1%) had moderate-or-greater PVL at predischarge. Among the 62 patients with initial moderate-or-greater PVL, 57 (91.9%) had regressed to mild or less at predischarge and only 5 (8.1%) remained moderate-or-greater, with no severe cases. PVL severity decreased significantly from immediately after deployment to predischarge (paired Wilcoxon signed-rank test, *P* < 0.001). The greater regression of PVL severity among patients who underwent balloon post-dilatation (48/50, 96.0%) vs. those who did not (9/12, 75.0%) was consistent with active intraprocedural management of a dynamic, deployment-related leak rather than a fixed defect.

Longer-term follow-up imaging was available for 59 of the 62 patients with initial moderate-or-greater PVL (follow-up ranging from 1 month to 3 years). At the latest available follow-up, 57 (96.6%) showed mild-or-less PVL and only 2 (3.4%) remained moderate, with no severe cases. Given the heterogeneous follow-up intervals and the possibility of selective follow-up bias, the longer-term data are presented as a descriptive observation only, and the primary conclusions of this study remain confined to initial post-deployment and predischarge PVL.

## 4 Discussion

### 4.1 Principal findings

This study evaluated the association between annular EI and initial post-deployment moderate-or-greater PVL after TAVR with the first-generation self-expanding VenusA valve in BAV patients. The main findings were: (1) although EI was modestly higher in patients with moderate-or-greater PVL, its stand-alone discrimination was poor (AUC = 0.625); (2) EI ≥0.19 nonetheless remained independently associated with the endpoint after adjustment and across sensitivity analyses; (3) adding EI improved model discrimination only marginally, which remained limited after optimism correction; and (4) semi-quantitative leaflet and raphe calcification were not independently associated with the endpoint in this cohort. Taken together, these findings position EI as a supplementary anatomic marker within integrated risk stratification rather than as a stand-alone predictive tool.

The numerically large but unstable odds ratio for age ≥65 years should not be interpreted substantively, as it reflects sparse-data bias rather than a true effect; this is confirmed by its marked attenuation under both Firth penalized regression and continuous-age modeling.

### 4.2 Annular morphology and the mechanism of PVL

Annular asymmetry is one of the core anatomical features that distinguish BAV from TAV. In patients with BAV, owing to the abnormal embryological development of leaflet fusion, the geometry of the annulus deviates markedly from circular in both diastole and systole, manifesting as an increased EI (9, 23). Previous studies have shown that each 0.1 increase in EI is associated with an approximately 40%–60% increase in the risk of PVL after self-expanding valve implantation (10). The mechanism can be explained as follows: an asymmetric annular geometry causes the radial force of the nitinol stent of a self-expanding valve to be distributed unevenly along the long axis during annular expansion, leading to a crescent-shaped paravalvular gap between the stent and the annulus and thereby increasing the risk of PVL. This geometric-mismatch mechanism is consistent with previous reviews of the multifactorial pathogenesis of PVL, in which PVL is determined by the interaction of multiple factors, including annular morphology, calcification distribution, and valve type (22). Similar associations were reported by Wong et al. (10) with the CoreValve and Jilaihawi et al. (11) with the SAPIEN valve, and Zito et al. (8) confirmed the self-expanding valve as an independent predictor of PVL in BAV (OR = 9.01). The present study extends this association to the Chinese self-expanding VenusA platform in a specifically BAV population.

The relatively high incidence of initial moderate-or-greater PVL in this study (32.0%) is substantially higher than the ∼4% reported for newer-generation valves in contemporary BAV registries (8), and this apparent discrepancy is explained by the timing of assessment rather than by a genuinely worse outcome. Crucially, initial post-deployment PVL is a pre-correction, pre-stabilization measurement and is not equivalent to final procedural PVL. Three factors account for the gap. First and most important, we graded PVL before any corrective maneuver, whereas most published incidences describe final or predischarge PVL recorded after correction; our endpoint therefore captures leaks still amenable to these maneuvers. Second, all patients received the first-generation skirtless VenusA valve, which lacks the outer sealing skirt that markedly reduces early PVL in newer systems. Third, the cohort consisted entirely of BAV patients, in whom pronounced annular eccentricity and heterogeneous calcification predispose to a transient crescentic annulus–stent gap at the moment of deployment.

Two lines of internal evidence reinforce that much of this initial PVL is dynamic rather than fixed. First, balloon post-dilatation was performed far more often in the initial moderate-or-greater PVL group (88.3% vs. 28.1%; Table 1)—a corrective response to, not a cause of, the leak—consistent with intraprocedural efforts to reduce these leaks. Second, and most importantly, moderate-or-greater PVL fell from 32.0% immediately after deployment to only 3.1% at predischarge, with 91.9% of affected patients regressing to mild or less (paired *P* < 0.001). Notably, this 3.1% predischarge rate is comparable to the ∼4% reported for newer-generation, skirted valves in contemporary BAV registries (8). This convergence indicates that the high initial incidence reflects the transient annulus–stent geometry at the moment of release rather than a genuinely inferior clinical result, and that the skirtless first-generation platform primarily amplifies—and thereby renders measurable—the underlying anatomical signal.

This trajectory also clarifies why studying the initial, pre-correction endpoint is meaningful rather than futile. We fully acknowledge that initial post-deployment PVL largely resolves and carries little independent long-term consequence. However, the low predischarge rate was not a spontaneous outcome but the product of active intraprocedural correction, as shown by the higher regression among post-dilated patients (96.0% vs. 75.0%). Initial post-deployment PVL is therefore best understood as an intervened-upon intermediate variable: its limited downstream effect exists precisely because it is routinely corrected, not because it is unimportant. Focusing on this specific time point offers two advantages that a final-PVL endpoint cannot. It is the only moment at which PVL is directly visible and modifiable by the operator, so that identifying anatomically high-risk patients preprocedurally is equivalent to anticipating who will require more aggressive intraprocedural management; and it is the only endpoint that isolates the pure annulus–frame geometric interaction, whereas final PVL is confounded by the very corrective maneuvers that mask the underlying anatomical signal. Furthermore, achieving this favorable final result appears not to be without cost: the reduction of PVL from 32.0% to 3.1% was accompanied by balloon post-dilatation in a substantial proportion of patients, a maneuver associated with well-recognized risks including annular injury, stroke, and conduction disturbance (25, 26). Preprocedural recognition of high annular eccentricity may thus prompt more deliberate initial positioning and oversizing, readiness for corrective maneuvers, and—where appropriate—consideration of an alternative platform, potentially reducing reliance on remedial post-dilatation. In this sense, the value of EI lies not in predicting long-term harm, but in informing the intraprocedural strategy, and the attendant costs, required to achieve a good final result.

### 4.3 Calcification and the sievers classification

Leaflet calcification is a well-established contributor to post-TAVR PVL (24). In the present cohort, however, neither visually graded moderate-or-greater leaflet calcification nor raphe calcification was *independently* associated with initial post-deployment PVL. This null finding must be interpreted as a limitation of the present measurement approach rather than as evidence that calcification is unimportant. Several factors plausibly attenuated a true association: visual grading cannot quantify calcium volume, density, asymmetry, or landing-zone distribution; BAV calcification is often focal and raphe-related, so a leaflet-level grade may misclassify the leak-relevant burden; and the restricted exposure range in this heavily calcified cohort (55.2% moderate-or-greater) further limits power. Accordingly, our results should not be read as showing that calcification has no role in PVL; rather, they indicate that visual grading was insufficient to isolate its contribution once annular eccentricity was accounted for. Quantitative CT calcium scoring, regional/asymmetry-resolved calcium mapping, and three-dimensional BAV morphology are needed to define this relationship.

In addition, the Sievers classification, which reflects raphe morphology, showed no significant association with initial post-deployment PVL (before corrective maneuvers) in this cohort (proportion of the raphe-containing type: 25.8% in the moderate-or-greater PVL group vs. 26.5% in the no/mild group; *P* = 1.000) (Supplementary Table S2). Across univariable, Sievers-adjusted, and fully adjusted models, the OR for EI ≥0.19 remained stable (2.98–3.01; all *P* ≤ 0.003), while Sievers classification showed no independent predictive value (all *P* > 0.50). This comparison suggests that, among the anatomical parameters examined here, the degree of annular geometric eccentricity was more informative than the qualitative morphological classification of the raphe, supporting the incorporation of the annular eccentricity index—a quantitative parameter—as one potentially important, supplementary component of integrated preprocedural assessment. Given the absence of implantation depth, quantitative calcium burden, and other relevant anatomical variables, eccentricity should be regarded as a contributory rather than a principal determinant of PVL.

### 4.4 Limitations

This study has the following limitations, which should be fully considered when interpreting the results.

First, this was a single-center retrospective study with a limited sample size and potential selection bias. In addition, immediate PVL was graded on-site by the TAVR team rather than by an independent blinded core laboratory; to limit this bias, grading followed VARC-3/ASE criteria, TEE served as the reference in discordant cases, and EI was measured offline independently of PVL grading. Although bootstrap internal validation was performed, external validation in an independent multicenter cohort is still required. Moreover, the 8-year enrollment period (2018–2025) may encompass an operator learning-curve effect and gradual refinements in imaging and grading practice, which were not adjusted for and may have introduced temporal heterogeneity in PVL assessment.

Second, the predictive performance was modest. EI alone yielded an AUC of only 0.625, and the optimism-corrected C-index of the model after adding EI was 0.609. Important determinants of PVL, including implantation depth, supra-annular/sealing-zone geometry, LVOT morphology, baseline left ventricular function, quantitative calcium burden, and standardized procedural risk variables, were not fully captured; the absence of these variables is itself a key reason we regard EI as a supplementary rather than an independent or dominant predictor, and it should be used only as one auxiliary parameter within a multidimensional assessment.

Third, calcification was assessed by visual semi-quantitative grading rather than quantitative CT calcium scoring, which (as detailed in Section 4.3) likely limited detection of an independent calcium effect despite excellent interobserver agreement.

Fourth, only traceable procedural variables, including valve size and balloon pre-/post-dilatation, were available. Valve implantation depth could not be reliably retrieved from the retrospective records and was not included in the model.

Fifth, valve-platform specificity. This study included only the first-generation self-expanding VenusA valve (a skirtless design), and extrapolation of the conclusions to newer-generation skirted self-expanding valves (e.g., VenusA-PRO and Evolut PRO/PRO+) or to balloon-expandable valves should be made with caution.

Sixth, the endpoint was initial post-deployment PVL assessed before corrective maneuvers. As detailed in Section 4.2, this endpoint is mechanistically informative but is not equivalent to final procedural, predischarge, 30-day, or long-term PVL, and its absolute incidence should not be compared directly with those benchmarks.

Seventh, baseline aortic regurgitation severity was not included. Although the cohort mainly consisted of patients with severe aortic stenosis and the endpoint was paravalvular rather than central regurgitation, baseline regurgitation remains a potential confounder and should be considered in future prospective studies.

Finally, the few patients with missing BMI were conservatively coded; a sensitivity analysis excluding them (Supplementary Table S6) confirmed that this did not affect the conclusions.

## 5 Conclusion

In patients with BAV undergoing TAVR with the first-generation self-expanding VenusA valve, higher annular eccentricity was independently associated with initial post-deployment moderate-or-greater PVL, whereas this initial leak largely resolved by discharge (from 32.0% to 3.1%) following intraprocedural correction. EI showed limited stand-alone discrimination, and the optimism-corrected performance of the multivariable model remained modest; the exploratory EI ≥0.19 threshold requires external validation and should not be used as a stand-alone clinical cutoff. EI should therefore be regarded as a supplementary anatomical dimension within integrated preprocedural risk assessment—one that may heighten anticipation of a challenging annulus–frame interaction and readiness for corrective maneuvers—rather than as an isolated decision-making criterion. The findings apply specifically to initial PVL after valve release and before post-dilatation and to this platform; extrapolation to final, 30-day, or long-term PVL, or to newer skirted or balloon-expandable valves, requires caution. Larger multicenter studies incorporating implantation depth, sealing-plane eccentricity, quantitative calcium burden, three-dimensional BAV morphology, and external validation are needed.

## Data Availability

The raw data supporting the conclusions of this article will be made available by the authors, without undue reservation.
