# Supplementary material for: Association between annular eccentricity and initial post-deployment paravalvular leak before corrective maneuvers in bicuspid aortic valve patients undergoing self-expanding transcatheter aortic valve replacement: a single-center retrospective study
Source: Front Cardiovasc Med. 2026 Aug 5;13:1909489. doi: 10.3389/fcvm.2026.1909489 (PMC13486284; doi:10.3389/fcvm.2026.1909489)
Supplement: Supplementary file 1 [file Supplementaryfile1.docx]

**Supplementary Table S1** Firth's penalized likelihood logistic regression for predictors of initial post-deployment moderate-or-greater paravalvular leak (sensitivity analysis addressing data sparsity).

| **Variable** | **OR (95% CI)** | **P value** |
| --- | --- | --- |
| Eccentricity index ≥ 0.19 | 2.82 (1.38–5.79) | 0.0046 |
| Moderate-or-greater calcification | 1.04 (0.53–2.01) | 0.9137 |
| Raphe calcification | 0.94 (0.43–2.07) | 0.8792 |
| Age ≥ 65 years | 4.93 (0.84–29.06) | 0.0777 |
| Male sex | 0.92 (0.48–1.78) | 0.8038 |
| NYHA class ≥ III | 1.59 (0.70–3.61) | 0.2625 |
| BMI ≥ 25 kg/m² | 0.99 (0.52–1.90) | 0.9821 |

*Footnote: Odds ratios (OR) with 95% confidence intervals (CI) are reported. n = 194; events (moderate-or-greater PVL) = 62. BMI, body mass index; EI, eccentricity index; NYHA, New York Heart Association.*

**Supplementary Table S2** Sensitivity analysis demonstrating the stability of the eccentricity index (EI) effect after adjustment for Sievers classification.

| **Model** | **EI ≥ 0.19 OR (95% CI)** | **P value** | **Sievers (Type 1/2) OR (95% CI)** | **P value** |
| --- | --- | --- | --- | --- |
| Model A: EI alone | 2.98 (1.45–6.11) | 0.0029 | — | — |
| Model B: EI + Sievers classification | 2.98 (1.45–6.11) | 0.0029 | 0.95 (0.47–1.91) | 0.8775 |
| Model C: Full multivariable model + Sievers | 3.01 (1.44–6.27) | 0.0033 | 0.62 (0.13–3.05) | 0.5575 |

*Footnote: All models are logistic regression models for initial post-deployment moderate-or-greater paravalvular leak (n = 194; events = 62). EI ≥ 0.19 was entered as a binary predictor; Sievers classification was dichotomized as type 0 versus type 1/2 (presence of a raphe). Model A includes EI alone; Model B additionally adjusts for Sievers classification; Model C is the full multivariable model (EI ≥ 0.19, age ≥ 65 years, male sex, BMI ≥ 25 kg/m², NYHA class ≥ III, moderate-or-greater calcification, and raphe calcification) with Sievers classification added. The odds ratio for EI ≥ 0.19 remained essentially unchanged across all models, whereas Sievers classification showed no independent association. CI, confidence interval; EI, eccentricity index; OR, odds ratio. Dashes (—) denote variables not entered into the corresponding model; Model A included EI alone.*

**Supplementary Table S3** Multivariable logistic regression with continuous EI (robustness analysis).

| **Variable** | **OR (95% CI)** | **P** |
| --- | --- | --- |
| Eccentricity index (per 0.1 increase) | 1.87 (1.08–3.25) | 0.026 |
| Moderate-or-greater calcification | 1.78 (0.65–4.88) | 0.259 |
| Raphe calcification | 0.86 (0.39–1.88) | 0.706 |
| Age ≥ 65 years | 7.63 (0.95–61.20) | 0.056 |
| Male sex | 0.94 (0.49–1.80) | 0.854 |
| NYHA class ≥ III | 1.62 (0.71–3.70) | 0.255 |
| BMI ≥ 25 kg/m² | 0.90 (0.47–1.73) | 0.749 |

*Footnote: Multivariable logistic regression with EI modeled as a continuous variable (odds ratio expressed per 0.1 increase). n = 194; events = 62. The direction and significance were consistent with the dichotomized model (EI ≥ 0.19). BMI, body mass index; EI, eccentricity index; NYHA, New York Heart Association.*

**Supplementary Table S4** Sensitivity analysis adding balloon predilatation to the primary multivariable model (*n* = 181, events = 60).

| **Variable** | **OR** | **95% CI** | **P** |
| --- | --- | --- | --- |
| EI ≥ 0.19 | 3.00 | 1.42–6.33 | 0.0039 |
| Heavy calcification | 0.99 | 0.48–2.05 | 0.9759 |
| Raphe calcification | 0.81 | 0.35–1.91 | 0.6353 |
| Age ≥ 65 | 7.53 | 0.92–61.70 | 0.0599 |
| Male | 1.13 | 0.56–2.28 | 0.7295 |
| NYHA III–IV | 1.89 | 0.80–4.48 | 0.1496 |
| BMI ≥ 25 | 0.98 | 0.50–1.92 | 0.9535 |
| Pre-dilatation (yes vs. no) | 1.31 | 0.47–3.64 | 0.6071 |

*Footnote: n = 181; events = 60. Five patients lacking measurable BMI (bedridden) were coded as BMI < 25, and 13 patients with missing balloon predilatation status were excluded (of whom 2 had moderate-or-greater PVL). Predilatation status was harmonized from two independent source datasets (concordance = 1.0). After additionally adjusting for balloon predilatation, the association between EI ≥ 0.19 and initial post-deployment PVL was essentially unchanged (OR 3.00; 95% CI 1.42–6.33; P = 0.0039), and predilatation itself was not associated with the outcome (OR 1.31; 95% CI 0.47–3.64; P = 0.607). BMI, body mass index; CI, confidence interval; EI, eccentricity index; NYHA, New York Heart Association; OR, odds ratio.*

**Supplementary Table S5** Multivariable logistic regression with age modeled as a continuous variable (Sensitivity analysis for Reviewer 1, minor comment 1; *n* = 194, events = 62).

| **Variable** | **OR** | **95% CI** | **P value** |
| --- | --- | --- | --- |
| Eccentricity index ≥ 0.19 | 2.97 | 1.43–6.16 | 0.0034 |
| Moderate-or-greater calcification | 1.04 | 0.53–2.02 | 0.919 |
| Raphe calcification | 1.03 | 0.47–2.28 | 0.938 |
| Age (per 1-year increase) | 1.02 | 0.98–1.07 | 0.292 |
| Male sex | 0.90 | 0.47–1.75 | 0.762 |
| NYHA class ≥ III | 1.56 | 0.69–3.55 | 0.290 |
| BMI ≥ 25 kg/m² | 1.04 | 0.55–1.99 | 0.898 |

*Footnote: When age was entered as a continuous variable instead of the ≥ 65-year dichotomy used in the primary model, the association between EI ≥ 0.19 and initial post-deployment moderate-or-greater PVL was essentially unchanged (OR = 2.97; 95% CI 1.43–6.16; P = 0.0034). Continuous age was not significantly associated with the outcome (OR = 1.02 per year; P = 0.292), confirming that the wide, unstable OR for dichotomized age ≥ 65 years in the primary model (OR = 7.30; 95% CI 0.91–58.39) reflected sparse-data bias rather than a true large effect. BMI, body mass index; CI, confidence interval; EI, eccentricity index; NYHA, New York Heart Association; OR, odds ratio; PVL, paravalvular leak.*

**Supplementary Table S6** Multivariable logistic regression excluding the five patients with missing BMI (Sensitivity analysis for Reviewer 1, minor comment 2; *n* = 189, events = 59).

| **Variable** | **OR** | **95% CI** | **P value** |
| --- | --- | --- | --- |
| Eccentricity index ≥ 0.19 | 2.81 | 1.34–5.89 | 0.0061 |
| Moderate-or-greater calcification | 1.03 | 0.52–2.04 | 0.932 |
| Raphe calcification | 0.91 | 0.40–2.04 | 0.809 |
| Age ≥ 65 years | 7.00 | 0.87–56.03 | 0.0667 |
| Male sex | 0.87 | 0.44–1.70 | 0.676 |
| NYHA class ≥ III | 1.61 | 0.70–3.71 | 0.263 |
| BMI ≥ 25 kg/m² | 1.03 | 0.53–2.00 | 0.932 |

*Footnote: The five bedridden patients with missing height/weight (patient IDs 38, 74, 110, 117, 164), who were conservatively coded as BMI < 25 kg/m² in the primary model, were excluded in this sensitivity analysis. EI ≥ 0.19 remained independently associated with initial post-deployment moderate-or-greater PVL (OR = 2.81; 95% CI 1.34–5.89; P = 0.006), confirming that the conservative handling of missing BMI did not materially affect the principal findings. Abbreviations as in Supplementary Table S5.*
